# Accuracy of digital impressions in edentulous arches for implant-supported prostheses: An *in vitro* comparison

**DOI:** 10.6026/973206300212996

**Published:** 2025-09-30

**Authors:** Ravi Ranjan Sinha, Poda Alekhya, Yalavarthy Prathyusha, Harprit Kaur, Krishnaprasad T.R., Biswajit Panda

**Affiliations:** 1 Department of Prosthodontics and Crown & Bridge, The Oxford Dental College, Bangalore, India; 2 Department of Prosthodontics and Crown & Bridge, The Oxford Dental College, Bengaluru, India; 3 Kalinga Institute of Dental Sciences, KIIT, Deemed to be university, Bhubaneswar, Odisha, India

**Keywords:** Digital impression, edentulous arch, implant-supported prosthesis, intraoral scanner, accuracy, 3D analysis

## Abstract

Accurate impressions are essential for the success of implant-supported prostheses, yet capturing edentulous arches remains challenging due to mucosal support and anatomical variability. This *in vitro* study compared the accuracy of conventional open-tray impressions with two intraoral scanners (IOS) in maxillary and mandibular edentulous models with six implant analogs. Ten impressions per group were analyzed by superimposing STL files and stone casts against a reference model to measure linear and angular deviations. Results showed significantly lower deviations in both digital groups compared to conventional impressions, with no significant difference between the two IOS types. These findings indicate that intraoral scanners provide superior accuracy and represent a clinically viable alternative for full-arch implant rehabilitations.

## Background:

The evolution of digital technology in prosthodontics has significantly transformed the process of impression making, especially for implant-supported prostheses [1]. Accurate impressions are fundamental for passive fit of the superstructure, which directly affects the long-term success of implant restorations [1]. Conventional impressions, although considered the gold standard, are technique-sensitive and susceptible to dimensional distortions due to factors such as material shrinkage, tray movement and stone expansion [2, 3]. Digital impressions using intraoral scanners (IOS) offer a non-invasive and efficient alternative by capturing 3D data directly from the oral cavity without the use of impression materials [4]. While IOS systems have demonstrated high accuracy in dentate arches, their performance in edentulous arches remains a subject of debate due to the lack of definitive anatomical landmarks and the increased difficulty in registering mucosal surfaces [5, 6]. Full-arch scanning also poses challenges with image stitching errors and scan distortions, particularly in the absence of fixed reference points like teeth [7]. Several *in vitro* studies have attempted to evaluate the trueness and precision of digital impressions in implant-supported restorations, showing promising results in partially edentulous conditions [8, 9]. However, limited data exist regarding their accuracy in completely edentulous arches where multiple implants are involved. Given the growing clinical preference for digital workflows, it is essential to validate their reliability under controlled conditions [5, 6]. Conventional impression techniques, particularly the open-tray method, have been widely accepted for recording implant positions with high accuracy. However, these methods are time-consuming and often uncomfortable for patients, especially those with edentulous arches. Additionally, dimensional inaccuracies can occur during impression material polymerization, tray removal, or stone cast fabrication, all of which compromise the precision of the final prosthesis [7]. Despite careful technique, discrepancies can arise, particularly in full-arch cases, leading to ill-fitting prostheses and biomechanical complications [8]. Digital impression systems, on the other hand, eliminate several error-prone steps inherent in traditional workflows. By capturing the implant scan bodies directly through optical scanning, intraoral scanners provide clinicians with immediate visual feedback and reduce chairside time [9]. This digital workflow not only enhances patient comfort but also streamlines communication between the clinic and laboratory. The ability to visualize and evaluate the scan data in real-time also allows for immediate corrections, which is not feasible with conventional materials. In edentulous cases, however, the lack of teeth and rigid intraoral structures presents a major limitation for digital scanners. Therefore, it is of interest to compare the accuracy of digital and conventional impression techniques for implant-supported prostheses in edentulous arches using a standardized *in vitro* model.

## Materials and Methods:

This *in vitro* study was conducted to evaluate and compare the accuracy of digital and conventional impression techniques for implant-supported prostheses in completely edentulous arches. Two identical edentulous maxillary and mandibular models made of polyurethane resin were fabricated, each embedded with six parallel implant analogs (three anterior and three posterior) positioned at predetermined locations.

## Three impression techniques were evaluated and divided into the following groups:

[1] Group A: Conventional open-tray impression using polyether impression material and splinted copings.

[2] Group B: Digital impression using Intraoral Scanner Type 1 (IOS-1).

[3] Group C: Digital impression using Intraoral Scanner Type 2 (IOS-2).

For Group A, custom trays were fabricated for both arches and open-tray impression copings were splinted using light-cured resin. Impressions were taken using medium-body polyether and poured with Type IV dental stone to obtain definitive casts. In Groups B and C, digital impressions were performed directly on the models using respective intraoral scanners according to the manufacturer's scanning protocols. All scans were saved in STL format and transferred to 3D analysis software. The reference model for each arch was obtained using an industrial-grade laboratory scanner with a resolution of 10 µm. Each impression or scan was superimposed on the reference model using best-fit alignment in reverse engineering software. Linear deviation (in microns) and angular discrepancy (in degrees) were measured at all implant sites. Each group included ten samples, and all measurements were performed by a blinded operator. Data were analyzed using SPSS software version 25.0. One-way ANOVA followed by Tukey's post hoc test was used to determine statistical significance among groups, with p < 0.05 considered significant.

## Results:

A total of 30 impressions (10 per group) were analyzed for linear deviation and angular discrepancy at six implant positions in each model. The results revealed that digital impression techniques (Groups B and C) showed superior accuracy compared to the conventional method (Group A). The mean linear deviation values at implant sites for each group are presented in Table 1 (see PDF). Group A exhibited the highest average deviation of 58.3 ± 12.4 µm, followed by 43.7 ± 10.9 µm in Group B and 39.2 ± 9.5 µm in Group C. The differences between Group A and the digital groups were statistically significant (p < 0.01), while the difference between Groups B and C was not significant (p > 0.05) (Table 1 - see PDF). Angular deviation analysis showed that the conventional method (Group A) had a mean deviation of 0.52° ± 0.13°, while digital impressions had lower values: 0.37° ± 0.10° for Group B and 0.34° ± 0.09° for Group C. Significant differences were noted between Group A and the digital groups (p > 0.05), with no significant variation between the two digital systems (Table 2 - see PDF). The trueness and precision values derived from the 3D overlay analysis are summarized in Table 3 (see PDF). Digital methods demonstrated higher trueness (lower deviation from the reference model) and precision (less variability) compared to the conventional method. These findings indicate that digital impression systems provide better accuracy and consistency in capturing implant positions in edentulous arches compared to conventional methods (Tables 1-3 - see PDF).

## Discussion:

The accuracy of impressions plays a pivotal role in the clinical success of implant-supported prostheses, especially in edentulous arches where mucosal support is minimal and implant position is critical. The present *in vitro* study compared conventional open-tray impressions with two different intraoral scanner systems for full-arch implant impressions and revealed that digital techniques provided superior trueness and precision. Our results are consistent with previous studies demonstrating the improved accuracy of digital impressions in capturing multiple implant positions [1, 2]. Both digital groups (B and C) showed significantly lower linear and angular deviations compared to conventional impressions. These findings support the premise that digital scanners reduce cumulative errors associated with multiple procedural steps, such as impression material distortion, cast pouring inaccuracies, and coping misalignments [3, 4]. Despite these advantages, digital impression techniques in edentulous cases still face challenges due to the absence of reliable anatomical landmarks. Full-arch scanning requires multiple image stitching steps, which can introduce cumulative errors if not managed correctly [5, 6]. However, recent technological advancements and software improvements have significantly enhanced the scanner's ability to recognize and align features, even in edentulous scenarios [7]. In the current study, both intraoral scanners demonstrated comparable outcomes, indicating that scanner type may have a limited impact on accuracy if proper scanning protocols are followed. This observation is in agreement with prior investigations that reported similar performance across different scanner models in complete-arch implant cases [8, 9]. Another noteworthy observation was the improved precision in digital groups, indicating better repeatability. Digital workflows reduce human-dependent errors by minimizing steps such as tray selection, impression removal, and material handling [10]. In contrast, conventional impressions remain technique-sensitive and operator-dependent, especially in complex cases like edentulous arches. Clinical relevance of digital accuracy is also highlighted in the literature, where minor discrepancies at implant positions may translate into biologic and mechanical complications such as screw loosening, component fracture, or marginal bone loss over time [11, 12]. Therefore, the enhanced accuracy of digital impressions could contribute to improved prosthetic fit and long-term success. However, some limitations must be acknowledged. This study was conducted *in vitro*, under controlled conditions without soft tissue mobility, saliva, or patient-related movement. In clinical settings, factors such as scanner access, patient cooperation, and tissue reflections may influence scanning quality [13]. Future clinical trials are necessary to validate these findings *in vivo*. Furthermore, cost and learning curve associated with digital systems may be a concern, especially in low-resource settings. Nonetheless, as technology becomes more accessible and user-friendly, digital impressions are likely to become the standard of care [14]. Conventional impressions demonstrated higher overall accuracy than digital impressions for full-arch implant-supported prostheses, although certain intraoral scanners such as the i500 showed comparable precision in specific parameters [15].

## Conclusion:

Digital impressions using intraoral scanners demonstrated superior accuracy and repeatability compared to conventional open-tray techniques in capturing implant positions for edentulous arches. The reduced linear and angular deviations in digital groups highlight the potential of intraoral scanning for full-arch implant-supported prostheses. With appropriate protocols, digital workflows can enhance clinical outcomes and streamline prosthetic procedures.
